# Dispersion of Tumors by Puncture

**Published:** 1874-11

**Authors:** 


THE DISPERSION OF TUMORS BY PUNCTURE.—Dr. Cameron, in the *Lancet*, observes that those familiar with the East are aware that, from time immemorial, the native hakims have been accustomed to attempt to bring about the absorption of the enlargements of liver and spleen, so common in hot, malarious countries, by the use of puncture with long, sharp stilets of considerable thickness. Twining, in his work "On the Diseases of Bengal," mentions the practice. Dr. Cameron states that he has never followed it for the purpose of procuring dispersions of such enlargements, but that he has frequently seen those of the liver disappear rapidly after repeated plunges of an ordinary hydrocele trocar, when seeking unsuccessfully for suspected abscess, and he never found in any instance inflammatory or any other bad symptoms produced by such operations, strange as it may appear to those unaccustomed to perform them. What he wishes particularly to draw attention to is, that other enlargements besides those of the liver and spleen may be made to disappear by puncture. Nothing is more tedious than those chronic glandular swellings which, in strumous subjects, often, in hot countries, follow upon trifling causes, such as angry mosquito bites, riding a rough bucking horse, over exertion, or a strain in cricketing, and so forth. He states that he has known an officer laid up for many months, and ultimately invalided, with a large mass of indurated enlarged glands, occupying the whole inguinal region, and resisting all the recognized routine of treatment. Accident showed him that deep puncture of such masses with a common lancet held at right angles to the swelling, and pushed down to its bottom, will often cause absorption to set in and proceed rapidly. The first case in which this occurred to him was one of a mercantile gentleman, disabled by a mass of swollen inguinal glands, almost as hard as board, and resisting all treatment. This patient's loss of time at the office was a very serious matter to him, and, influenced by his despairing impatience, Dr. Cameron plunged a lancet perpendicularly into the mass as far as it would reach. The point came out tinged with matter, and hard pressure brought up a little cheesy, ill-formed pus, but no discharge whatever followed, and absorption set in and proceeded rapidly. This and several other cases which he mentions, have led him to think that this mode of puncture might be found to bring about the dispersion of such growths as fibrous tumors of the uterus; and reasoning from the non-supervention of evil symptoms after repeated and deep puncture of the liver with a trocar, he sees no ground for fearing to puncture with a small stilet such a fibrous uterine tumor as is often plainly to be felt through the abdominal parietes, and he thinks puncture through them less likely to be followed by evil consequences than puncture per vaginam, owing to the exclusion of air.

